# Novel nanoconjugates of metal oxides and natural red pigment from the endophyte *Monascus ruber* using solid-state fermentation

**DOI:** 10.1186/s12934-024-02533-8

**Published:** 2024-09-29

**Authors:** El-Sayed R. El-Sayed, Gharieb S. El-Sayyad, Sobhy S. Abdel-Fatah, Ahmed I. El-Batal, Filip Boratyński

**Affiliations:** 1 Department of Food Chemistry and Biocatalysis, Wrocław University of Environmental and Life Sciences, Norwida 25, 50-375 Wrocław, Poland; 2 Plant Research Department, Nuclear Research Center, Egyptian Atomic Energy Authority, Cairo, Egypt; 3 Drug Radiation Research Department, National Center for Radiation Research and Technology (NCRRT), Egyptian Atomic Energy Authority (EAEA), Cairo, Egypt

**Keywords:** Red pigment, *Monascus ruber*, Metal oxide, Antimicrobial, Nano-conjugates, Antibiofilm

## Abstract

**Background:**

Antimicrobial resistance has emerged as a major global health threat, necessitating the urgent development of new antimicrobials through innovative methods to combat the rising prevalence of resistant microbes. With this view, we developed three novel nanoconjugates using microbial natural pigment for effective application against certain pathogenic microbes.

**Results:**

A natural red pigment (RP) extracted from the endophyte* Monascus ruber* and gamma rays were applied to synthesize RP-ZnO, RP-CuO, and RP-MgO nanoconjugates. The synthesized nanoconjugates were characterized by different techniques to study their properties. The antimicrobial potential of these nanoconjugates was evaluated. Moreover, the antibiofilm, protein leakage, growth curve, and UV light irradiation effect of the synthesized nanoconjugates were also studied. Our results confirmed the nano-size, shape, and stability of the prepared conjugates. RP-ZnO, RP-CuO, and RP-MgO nanoconjugates showed broad antimicrobial potential against the tested bacterial and fungal pathogens. Furthermore, the RP-ZnO nanoconjugate possessed the highest activity, followed by the RP-CuO against the tested microbes. The highest % inhibition of biofilm formation by the RP-ZnO nanoconjugate. Membrane leakage of *E. coli* and *S. aureus* by RP-ZnO nanoconjugate was more effective than RP-MgO and RP-CuO nanoconjugates. Finally, UV light irradiation intensified the antibiotic action of the three nanoconjugates and RP-ZnO potential was greater than that of the RP-MgO, and RP-CuO nanoconjugates.

**Conclusion:**

These findings pave the way for exploiting the synthesized nanoconjugates as potential materials in biomedical applications, promoting natural, green, and eco-friendly approaches.

**Supplementary Information:**

The online version contains supplementary material available at 10.1186/s12934-024-02533-8.

## Introduction

Antimicrobial resistance is a pressing global health concern, intensified by the misuse and overuse of antibiotics [1]. As one of the main causes of mortality and morbidity, it increases the economic burden on health systems [2]. In 2019, infections by drug-resistant bacteria resulted in 1.27 million deaths [3, 4]. Without intervention, this number is expected to reach 10 million deaths annually by 2050 [1, 5]. Biofilms present a major challenge in treating bacterial infections and are a key factor in the persistence of these infections [6]. A biofilm contains organized layers of bacterial growth attached to the surface and encased in the extracellular polymeric matrix. Within a biofilm, microbial cells are shielded from the immune response and are up to 1000 times more resistant to antibiotics than their free counterparts [7, 8]. Despite the increasing threat of antimicrobial resistance, introducing new antibiotic classes since 2000 has been alarmingly low [9]. Obstacles to developing new antibiotics through traditional financing models include low returns on investment, risks for developers [10], and scientific challenges, such as difficulties in isolating new bioactive compounds with antimicrobial and antibiofilm potentials [9]. Consequently, there is an urgent social and scientific need to develop innovative technologies for new antimicrobials.

An alternative to the costly and lengthy process of de novo antimicrobial discovery is the development of novel ones by repurposing existing bioactive natural products using innovative formulations [11] such as nanoconjugation with metal nanoparticles [4]. The integration of nanotechnology and biotechnology offers endless possibilities and the potential to address various biological challenges [12] by enhancing the antimicrobial spectrum, reducing the need for high doses, and extending their longevity [13]. In particular, the use of microbial platforms is a promising alternative to develop an easy, rapid, and green factories for production of several types of nanomaterials. Generally, metal nanoparticles, known for their broad antimicrobial spectrum and large surface area, are particularly promising for conjugation. Metal oxide nanoparticles (NPs), such as CuO [14], MgO [15], and ZnONPs [16], are especially effective as antimicrobials and antibiofilm agents due to their physicochemical properties. Recently, studies have suggested that conjugating nanoparticles with antimicrobials can enhance antimicrobial activity [11]. For example, the conjugation of bacteriocin antibiotics with nanoparticles has shown improved efficacy against certain food-spoiling organisms and drug-resistant pathogens [13]. In the same connection, *Monascus* fungi, well-known in Asian countries for centuries, produce red pigments with various therapeutic applications such as antimicrobial, immunosuppressive, antioxidant, anti-tumor, and cytotoxic properties [17]. Moreover, these red pigments are effective against several bacterial strains and some filamentous fungi [18–20]. Therefore, developing such nanoconjugates as antimicrobial tools through green methods holds significant potential for pharmaceutical and food industry applications. This study reports, for the first time, the synthesis of novel red pigment and metal oxide nanoconjugates. The antimicrobial activity of these synthesized conjugates against various bacterial and fungal pathogens was evaluated. Additionally, the study examined the antibiofilm effects, bacterial protein leakage, and antibacterial activity induced by photo-generated reactive oxygen species of the three nanoconjugates under UV light irradiation.

## Materials and methods

### Chemicals and reagents

Zn(NO_3_)_2_⋅6H_2_O, CuSO_4_ (anhydrous), and MgSO_4_ (anhydrous) were procured from Sigma Aldrich (St. Louis, MO, USA). The microbiological testing media (nutrient broth, nutrient agar, malt extract agar.) were supplied from Oxoid (UK). *Monascus* pigments authentic standards (Monascorubramin, Rubropunctamine, Monascorubrin, Rubropunctatin, and Monascin) were purchased from Biosynth Ltd, UK.

### Fungal strain

*Monascus ruber* SRZ112 (GenBank accession number MT140350.2), was used as a source of the red pigment (RP) in this study [21]. The fungus was preserved in the culture collection of Assiut University Mycological Center (deposition number AUMC13976), Assiut, Egypt.

### Production, purification, and chemical characterization of RP

Production of the RP was achieved using solid-state cultivation according to a previous report [21], as follows; in a 250 mL Erlenmeyer flask 15 g potato peel was moistened by Mineral Salt Broth (pH 6.0) to attain a moisture content of 70%, w/w and autoclaved. Then, 1 mL of 7-day-old spore suspension (10^7^ spore mL^−1^) was added to the flask, mixing the contents thoroughly, and dark-incubated for 10 days at 25 °C. At the end of the incubation period, the whole culture was extracted by absolute ethanol and agitated (200 rpm) for 4 h. The mixture was filtered through Whatman paper No. 1 and centrifuged (5810R, Eppendorf) for 10 min. The resultant supernatant was added to silica gel (Silica gel 60, 0.040–0.063 mm, Merck, Germany). The whole solvent was evaporated on a vacuum evaporator. Pre-column was filled to 3/4 with silica gel and then, silica gel with fungal extract was added. The equipment used for purification was puriFlash XS520Plus (Interchim SA, France) with the column SIHP-JP, F0012, and *n*-hexane: ethyl acetate as an eluent. Purification started with 100% *n*-hexane, and then gradually the ratio of eluents changed for example 99% n-hexane and 1% ethyl acetate, then 98% *n*-hexane and 2% ethyl acetate (the polarity of the eluent increased). The final ratio of n-hexane: ethyl acetate was 95:5.

The separated RP was subjected to LC/ESI–MS/MS analysis to identify the chemical structure by comparison with standards. LC/ESI–MS/MS measurements were performed under the conditions previously described [21] using a RSLC Dionex UltiMate 3000 (Thermo Fisher Scientific, USA) with mass spectrometer ESI-Q-TOF, maXis impact (Bruker, USA) with Syncronis Phenyl C18 1.7 µm, 100 × 2.1 mm column (Thermo Scientific, USA). The separated RP was identified as rubropunctamine (Supplementary Fig. 1).

### Synthesis of RP-ZnO, RP-CuO, and RP-MgO nanoconjugates

A volume of 10 mL of 2.0 mM aqueous solution of Zn (NO_3_)_2_⋅6H_2_O, CuSO_4_, and MgSO_4_ was mixed separately with 80 mL of the separated RP (10 mg mL^−1^) at room temperature for 30 min. The pH of the mixtures was 7.2. The mixtures were kept at 30 °C for 24 h under continuous agitation. The three mixtures were irradiated by gamma rays at 20 kGy dose. Gamma irradiation was performed at the National Center for Radiation Research and Technology (Cairo, Egypt) at an exposure rate of 0.594 kGy per hour, using a ^60^Co Gamma chamber (4000-A, India). After irradiation, a color shift of the mixtures was observed, verifying the synthesis of RP-ZnO, RP-CuO, and RP-MgO nanoconjugates.

### Characterization of the synthesized nanoconjugates

Shape and the mean and accurate size of the synthesized RP-ZnO, RP-CuO, and RP-MgO were studied using HR-TEM (JEM2100, Jeol, Japan). The surface appearance and shape of the synthesized RP-ZnO, RP-CuO, and RP-MgO nanoconjugates were studied using SEM (ZEISS, EVO-MA10, Germany). The optical properties of the synthesized RP-ZnO, RP-CuO, and RP-MgO were recorded in the range of 190 and 900 nm using a UV–Vis spectrophotometer (JASCO V-560). X-ray diffraction analysis of the synthesized nanoconjugates was used to determine the crystalline nature and the crystal size of the synthesized using XRD-6000, Shimadzu Scientific Instruments, Japan (Cu-Kα target and nickel filter. Working by a Cu anode at 50.0 mA and 40.0 kV in the state of 2θ value inside 20° and 100° with a flow of 2°/min), adjusted with the XRD-6000 lists (including outstanding austenite quantitation, crystallinity estimation, stress examination, and crystallite size/lattice strain matters). Particle size distribution and zeta potential of synthesized nanoconjugates were recorded using DLS-PSS-NICOMP 380, USA. Fourier transform infrared (FT-IR) transmission spectra of synthesized RP-ZnO, RP-CuO, and RP-MgO were recorded (400 to 4000 cm^−1^) using a FT-IR Nicolet 6700 spectrometer.

### Testing the antimicrobial potential of the synthesized nanoconjugates

The synthesized RP-ZnO, RP-CuO, and RP-MgO nanoconjugates and RP samples were separately tested for their antimicrobial potential using the agar-well diffusion method [22]. The antibacterial activity of the synthesized RP-ZnO, RP-CuO, and RP-MgO nanoconjugates was evaluated against different types of Gram-positive and Gram-negative bacteria (Table 1) meanwhile the antifungal was evaluated against *Candida tropicalis* and *Candida albicans*. Microbial strains were kindly provided by the culture collection of the Drug Microbiology Lab (NCRRT, Cairo, Egypt). The microbial inoculums were standardized 2–5 × 10^8^ CFU mL^−1^ for bacteria and 1–4 × 10^8^ CFU mL^−1^ for the tested yeasts. The tested bacteria were inoculated on nutrient agar, while *Candida tropicalis* and *Candida albicans* were inoculated on malt extract agar. Positive standards of Amoxicillin (AX) and Nystatin (NS) were used to evaluate the ZOI potency. The inoculated plates were incubated at 37 °C for 24 h and the zone of inhibition (ZOI) was then measured.

**Table 1:** Crystallographic data and DLS analysis of the synthesized RP-ZnO, RP-CuO, and RP-MgO nanoconjugates

Parameter	Synthesized nanoconjugates		
	RP-ZnO	RP-CuO	RP-MgO
Crystal system	Hexagonal	Hexagonal	Hexagonal
Lattice parameter (nm)	0.634566	0.456770	0.343333
Mean crystallite size (nm)	33.20	30.30	31.85
Size distribution (nm)	321.0	210.58	152.52
Average size (nm)	48.61	42.60	43.65
Zeta potential (mV)	− 25.50	− 22.78	− 23.78
Polydispersity index	0.038	0.040	0.041

The mean crystallite size was calculated from the Scherrer equation. Average size, size distribution, zeta potential, and Polydispersity index were obtained from the DLS analysis as described in Materials and Methods

### Testing the antibiofilm potential of the synthesized nanoconjugates

The synthesized RP-ZnO, RP-CuO, and RP-MgO nanoconjugates were tested for their antibiofilm activities against chosen bacteria and *Candida* spp. using qualitative tests [23]. The microbial inoculums were prepared as mentioned previously, and then 5.0 mL nutrient broth was mixed separately with the RP-ZnO, RP-CuO, and RP-MgO nanoconjugates inside distinct test tubes. All the tubes’ components from the treated (with nanoconjugates) and control (cell-free supernatant) were discarded after incubation. After this, test tubes were washed with phosphate buffer saline (pH 7.0) followed by deionized water [24]. Bacterial cells inside tube walls were fixed with sodium acetate (5.0 mL, 3.5%) for 20 min, and washed with de-ionized water. In the case of forming a biofilm, it was stained with crystal violet (0.15%) and washed with de-ionized water. Finally, 5.0 mL ethanol (100%) was added and the optical density (OD) was recorded at 570 nm. The microbial biofilm inhibition percentage was calculated using the following equation:

$${\text{Percentage}}\;{\text{of}}\;{\text{biofilm}}\;{\text{inhibition}}\left( \% \right) = \left[ {\left( {{\text{OD}}_{{{\text{control}}}} - {\text{OD}}_{{{\text{treated}}}} } \right)/\left( {{\text{OD}}_{{{\text{control}}}} } \right)} \right] \times {1}00$$

### Effect of the synthesized nanoconjugates on the bacterial growth curve

The influence of synthesized RP-ZnO, RP-CuO, and RP-MgO nanoconjugates on the growth of the most sensitive bacterial strains (*S. aureus* and* E. coli*) was evaluated by the growth curve assay [25]. The bacterial suspension (1 × 10^8^ CFU mL^−1^) was added to tubes containing 5.0 mL of nutrient broth. The synthesized RP-ZnO, RP-CuO, and RP-MgO nanoconjugates were added separately to the tubes. The bacterial growth expressed as OD units (600 nm) was recorded every 2 h.

### Effect of the synthesized nanoconjugates on bacterial protein leakage

The bacterial culture of *S. aureus* and *E. coli* (18 h, 10^8^ CFU mL^−1^) and the synthesized nanoconjugates were mixed with 10 mL nutrient broth. Simultaneously, bacterial cultures were mixed with nutrient broth served as the control. Samples were incubated for 5 h at 37 °C then centrifuged for 15 min at 5000 rpm. Finally, Bradford reagent (1 mL) was added to the resultant supernatants (100 μL). After 10 min of dark incubation, the OD values (595 nm) were measured [26, 27].

### Effect of UV light irradiation on the antibacterial activity of the synthesized nanoconjugates

Bacterial cultures (0.5 McFarland, 10^8^ CFU mL^−1^), were incubated for 2 h. The synthesized RP-ZnO, RP-CuO, and RP-MgO nanoconjugates were placed within the predicted tubes. The antibacterial efficiency was assessed against *S. aureus* and *E. coli* by the optical density approach after UV light irradiation, as opposed to the non-UV reference irradiation [28]. Two sets of tubes were used: one set with the synthesized RP-ZnO, RP-CuO, and RP-MgO nanoconjugates that weren't exposed to UV light, while the other included them. The RP-ZnO, RP-CuO, and RP-MgO nanoconjugates were subjected to different exposure times of 0, 15, 30, 45, 60, and 75 min. The intensity was estimated as 6.9 mW cm^−2^ disturbance on the samples at 37 °C. At 600 nm, the samples' turbidity was measured, and the percent of inhibition was calculated by the following equation:

$${\text{Bacterial}}\;{\text{inhibition}}\left( \% \right) = \left[ {\left( {{\text{OD}}\;{\text{control}} - {\text{OD}}\;{\text{treated}}} \right)/\left( {{\text{OD}}\;{\text{control}}} \right)} \right] \times {1}00$$

### Statistical analysis

Statistical investigation of the obtained results was performed using ONE-WAY ANOVA (*P* < *0.05*) and arranged as Duncan’s multiple sequences using SPSS software (version 15, IBM, USA).

## Results and discussion

### Synthesis of RP-ZnO, RP-CuO, and RP-MgO nanoconjugates

Here, gamma radiation is the reducing agent of aqueous salts resulting in the corresponding metal oxides viz., ZnO, CuO, and MgO. Meanwhile, RP separated from the fungal culture acted as a capping agent to the formed oxides resulting in RP-ZnO, RP-CuO, and RP-MgO nanoconjugates. In the literature, gamma radiation was used for the reduction and preparation of nanomaterials due to the advantage of producing high-reducing electrons in the solution, without generating any byproducts [29]. Furthermore, the use of microbial platforms for nanomaterials’ synthesis has emerged as a green alternative for the physical and chemical approaches taking the advantages of the fungi biofactories including tolerance to improvement and the large-scale production [30].

### Morphology studies of RP-ZnO, RP-CuO, and RP-MgO nanoconjugates

**Fig. 1:**
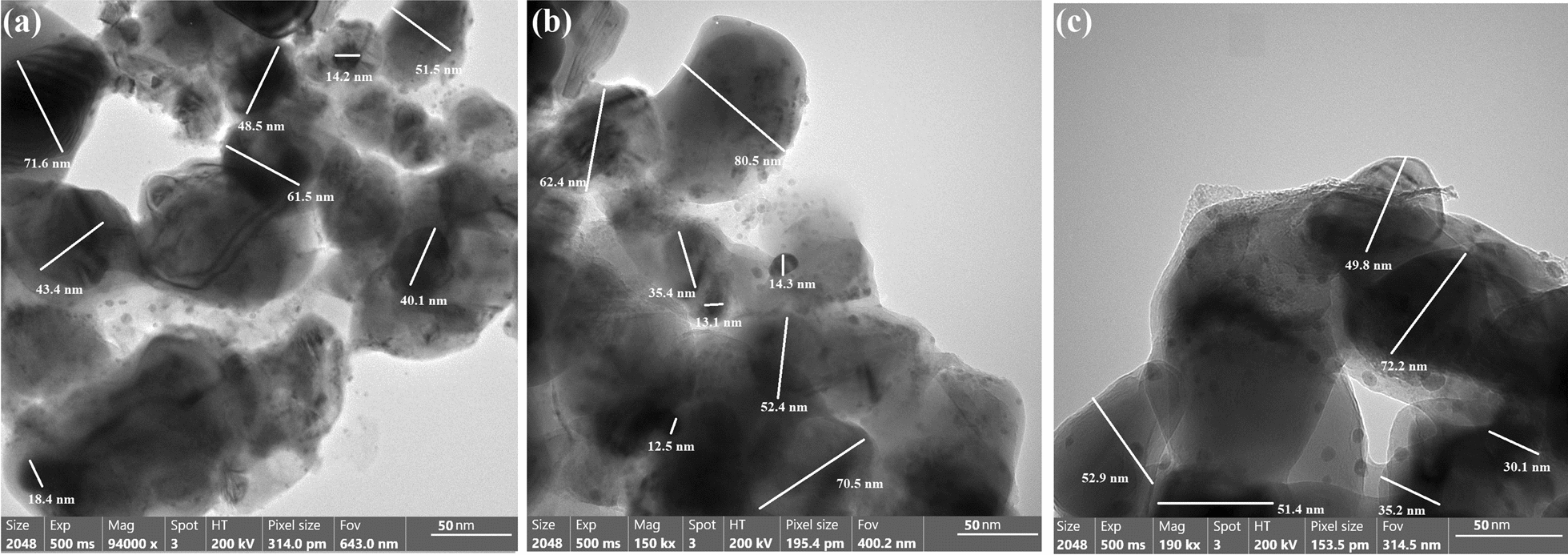
HR-TEM images of the synthesized RP-MgO (**a**), RP-CuO (**b**), and RP-ZnO (**c**) nanoconjugates

**Fig. 2:**
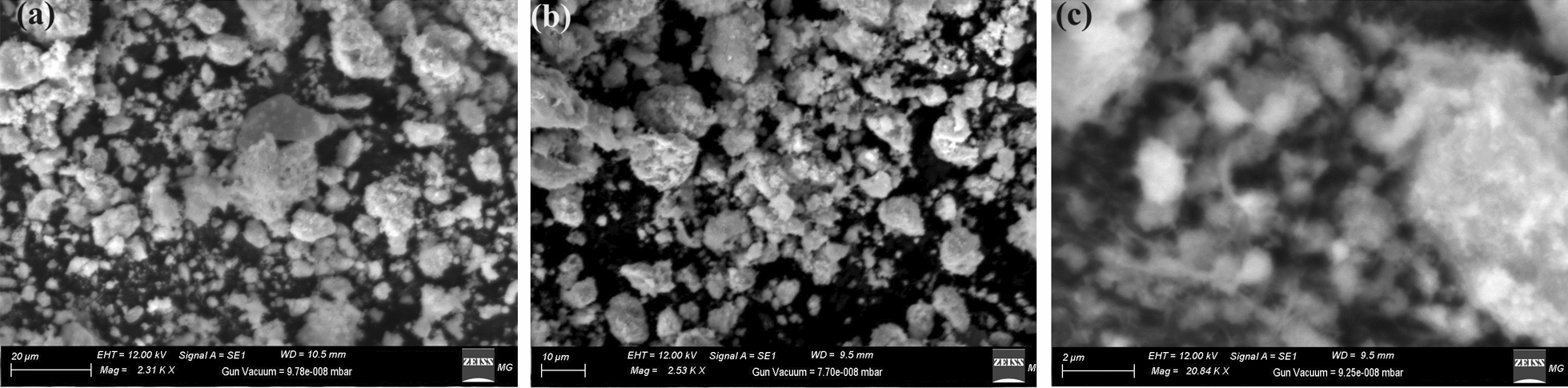
SEM images of the synthesized RP-MgO (**a**), RP-CuO (**b**), and RP-ZnO (**c**) nanoconjugates

To reveal the size and shape of the synthesized RP-ZnO, RP-CuO, and RP-MgO nanoconjugates, HR-TEM imaging was applied. Figure 1a presents the spheroidal shapes and mono-dispersity of the RP-MgO nanoconjugate with typical diameters that ranged from 14.2 to 71.6 nm and an estimated mean diameter of 43.65 ± 1.0 nm. The synthesized RP-CuO nanoconjugate was round in shape with an average diameter of 42.6 ± 1.2 nm and a size range of 12.5 nm to 80.5 nm (Fig. 1b). The synthesized RP-ZnO nanoconjugate HR-TEM images in Fig. 1c show a spherical shape with a diameter range of 30.1–72.2 nm and a mean diameter of 48.6 ± 1.4 nm. Furthermore, the shape and surface properties of the synthesized nanoconjugates were studied using SEM. Figure 2a presents the synthesized RP-CuO nanoconjugate as an isolated, rounded, bright particle on the fungal RP. In Fig. 2b, RP-MgO nanoconjugate also showed a similar pattern. Figure 2c presents a consistent RP-ZnO surface with a clear appearance as spherical aggregates merged throughout the RP's surface.

### Optical properties of RP-ZnO, RP-CuO, and RP-MgO nanoconjugates

**Fig. 3:**
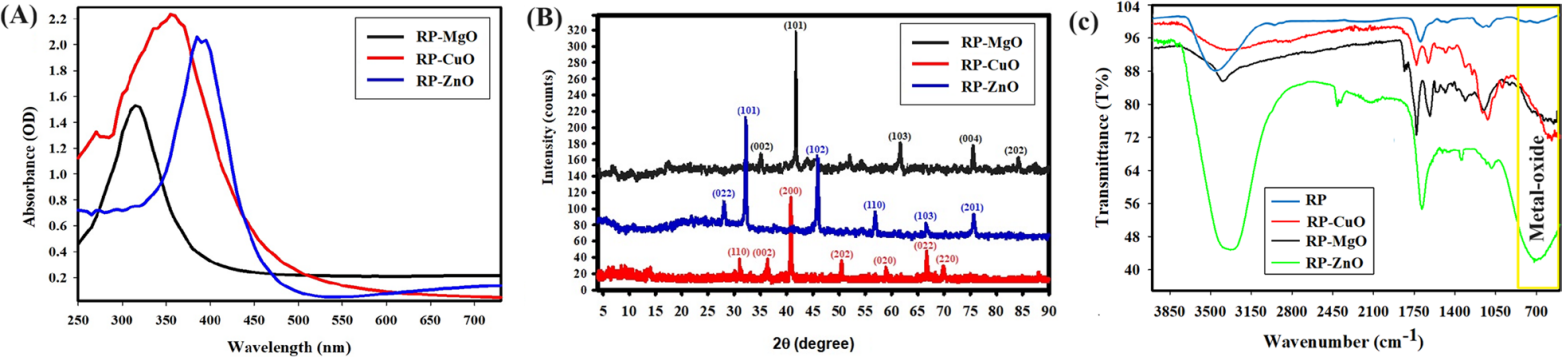
UV–Vis spectra (**a**), XRD patterns (**b**), and FTIR spectra (**c**) of the synthesized RP-MgO, RP-CuO, and RP-ZnO nanoconjugates

When RP-ZnO, RP-CuO, and RP-MgO were created, a color change was observed in the prepared solution's hue due to the stimulation of the SPR of the synthesized nanoconjugates [31]. Figure 3a presents the recorded UV maximum peaks for each nanoconjugate. The recorded experimental peaks were visible in the spectra at 360 nm (for RP-CuO), 315 nm (for RP-MgO), and 395 nm (for RP-ZnO). In partial agreement with previous work concerning the synthesis of CuONPs [14], MgONPs [15], and ZnONPs [16, 32] due to variations in the synthetic routes. Generally, SPR is greatly influenced by the durability, dimensions, morphology of the surfaces, construction, and dielectric characteristics of any produced nanoparticles [33–35].

### DLS analyses of RP-ZnO, RP-CuO, and RP-MgO nanoconjugates

According to DLS measurements, the particle size distribution of the synthesized RP-MgO, RP-CuO, and RP-ZnO nanoconjugates were 152.52, 210.58, and 321.0 nm, respectively (Table 1). Our results revealed that the PDI values for RP-ZnO, RP-CuO, and RP-MgO nanoconjugates were 0.041, 0.040, and 0.038, respectively. When the PDI value is less than 0.05, samples are said to be monodisperse according to the International Standards Organizations [36]. Our findings showed that the estimated sizes of the synthesized nanoconjugates according to HR-TEM imaging were smaller than those obtained from the DLS analysis. A plausible explanation for the noticed difference is the including the hydrodynamic radius inside the produced RP-ZnO, RP-CuO, and RP-MgO nanoconjugates and the water layers around them [37].

### XRD analysis of RP-ZnO, RP-CuO, and RP-MgO nanoconjugates

Here, XRD studies for the synthesized RP-ZnO, RP-CuO, and RP-MgO nanoconjugates are displayed in Fig. 3b and Table 1. XRD patterns of the three nanoconjugates contained amorphous (corresponding to the RP) and crystallographic (ZnONPs, CuONPs, and MgONPs) configurations. It is important to note that 2θ denotes the RP in the 2θ range from 5° to 21° [38]. The XRD findings of RP-ZnO nanoconjugate diffraction peaks are shown in Fig. 3b. These peaks at 2θ = 27.40°, 31.22°, 45.54°, 56.56°, 67.17°, and 75.56° are in agreement with JCPDS number 361451 and match the (002), (101), (102), (110), (103), and (201) Bragg's reflections [39]. Along with the typical card JCPDS number 892531, they additionally include the RP-CuO nanoconjugate diffraction peaks at 2θ = 30.22°, 36.11°, 40.75°, 52.72°, 58.27°, 67.83°, and 71.45°. These peaks match Bragg's reflections at degrees (110), (002), (200), (202), (020), (022), and (220) [40]. Finally, the XRD findings of RP-MgO nanoconjugate’s diffraction peaks were detected at 2θ = 35.20°, 41.25°, 61.90°, 75.21°, and 84.50°, which are complemented with a usual card JCPDS number 87-0653, match the (002), (101), (103), (004), and (202) Bragg’s reflections [41]. The produced RP-ZnO, RP-CuO, and RP-MgO nanoconjugates were crystallized and possessed a face-centered cubic (fcc) crystalline structure, according to the recorded XRD data (Table 1). Based on XRD data, highly crystalline ZnO, CuO, and MgONPs were produced and linked with amorphous RP, enhancing their dispersion in the solution for enhanced application [42]. Ultimately, the midway crystallite size of RP-ZnO, RP-CuO, and RP-MgO nanoconjugates was determined using the Williamson-Hall equation [43], and it was found to be 22.25, 28.36, and 30.25 nm.

### FT-IR spectroscopy and proposed synthetic mechanism

In this study, the conjugation between the RP and the synthesized ZnO, CuO, and MgO was studied by FT-IR. Figure 3c presents the recorded spectra of the RP and the synthesized nanoconjugates. All spectra of the RP-ZnO, RP-CuO, and RP-MgO nanoconjugates (Fig. 3c) and RP main bands corresponding to phenols, symmetric and asymmetric vibrations of C–O and C=O bonds in COO– group, OH in water, and C–H in CH_2_ and in the phenyl ring. However, spectra of the nanoconjugates only showed the formation of new bands at 588 cm^−1^ (RP-MgO), 602 cm^−1^ (RP-CuO), and 719 cm^−1^ (RP-ZnO) which are attributed to the metal–O interaction [44, 45], and the current findings align with Zhang et al.’s [46] explanation of the function of *Monascus* pigment in the environmentally friendly production of Ag NPs and the appearance of a particular absorption band at 2853.77 cm^−1^ in the produced Ag NPs spectra, which is caused by the carboxylic acid dimer formation.

**Fig. 4:**
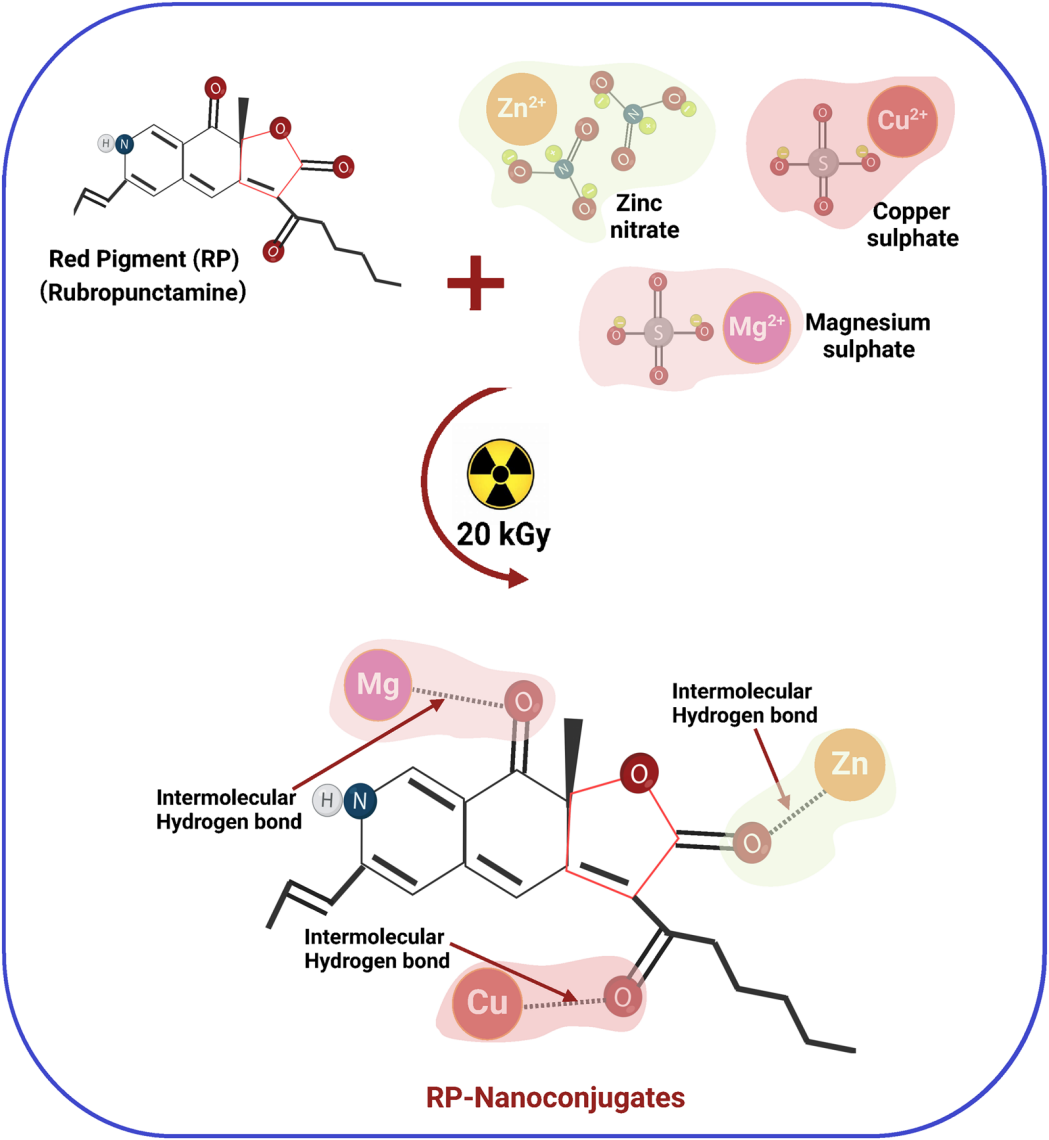
Schematic representation of the synthesis mechanism of the RP-MgO, RP-CuO, and RP-ZnO nanoconjugates. The figure was created in BioRender.com

Figure 3c further shows a peak in the RP spectrum at 3402 cm^−1^ due to the H–O–H stretching vibrations. These peaks were broadened at 3402, 3403, and 3344, cm^−1^ for RP-MgO, RP-CuO, and RP-ZnO, respectively, indicating the formation of intermolecular hydrogen bonding between RP, and the metal oxides [47]. Accordingly, FTIR results and the schematic representation in Fig. 4 of the synthesis mechanism of the RP-MgO, RP-CuO, and RP-ZnO nanoconjugates suggest gamma radiation acted as the reducing agents of aqueous salts resulting in the corresponding metal oxides viz., ZnO, CuO, and MgO. Then, RP acted as a capping agent to the formed oxides resulting in RP-ZnO, RP-CuO, and RP-MgO nanoconjugates.

The ability of extracts to synthesize extremely active metal oxide (Co_3_O_4_) NPs has been demonstrated by recent investigations, and the synthetic process is explained by FTIR data. Following Sidorowicz et al. [48] synthesis, the strength of all measured observed peaks with visible redshift decreased in the *Spirulina platensis* extract. Less of a drop was seen in the instances of *Chlorella vulgaris*, and *Haematococcus pluvialis*, and a redshift of around 3000 cm^−1^ was noted, which was ascribed to the proteins in the NH group. Overall, the findings support a major role for extract metabolites; primarily proteins and carbohydrates in the formation of Co_3_O_4_ NPs.

### Antimicrobial activity of RP-ZnO, RP-CuO, and RP-MgO nanoconjugates

It was found that certain biocidal agents were employed to prevent microbial diseases, but over time, the microbes developed resistance [49]. Consequently, new materials and methods were utilized to develop novel antimicrobials, especially those aimed at controlling the spread of diseases [50]. In this study, the antimicrobial activities of the synthesized RP-ZnO, RP-CuO, and RP-MgO nanoconjugates and the separated fungal RP were evaluated by agar well diffusion assay. Our results showed (Table 2) that the synthesized RP-ZnO, RP-CuO, and RP-MgO nanoconjugates showed a positive potency against a wide spectrum of bacteria. However, the fungal RP had the lowest recorded activities. In addition, RP-ZnO nanoconjugate showed the highest potential against *S. aureus*, *P. aeruginosa,* and *C. albicans*. Meanwhile, the antimicrobial potentials of RP-CuO, and RP-MgO were less than RP-ZnO against all the tested microbes (Table 2). Compared to the standard antibiotics (AX & NS), the synthesized RP-ZnO, RP-CuO, and RP-MgO nanoconjugates were more influential than all. The exact antimicrobial reaction mechanisms of the synthesized nanoconjugates are still not detected. In the literature, several mechanisms were proposed including changing the microbial membrane permeability [51, 52], changing the cell morphology and coating network, and initiating intracellular oxidative pressure due to the formation of H_2_O_2_ [53], diffusion of the reactive oxygen species [54], and the alkaline attraction [55].

**Table 2:** Antibacterial and antifungal potential of RP and the synthesized RP-ZnO, RP-CuO, and RP-MgO nanoconjugates

Tested pathogens	RP		RP-MgO		RP-CuO		RP-ZnO		AX and NS
	ZOI (mm)	MIC (µg/mL)	ZOI (mm)	MIC (µg/mL)	ZOI (mm)	MIC (µg/mL)	ZOI (mm)	MIC (µg/mL)	
Gram-positive bacteria									
*S. aureus*	9.5 ± 0.14	12.5	22.0 ± 0.15	0.195	21.5 ± 0.80	0.195	27.5 ± 0.50	0.048	7.2 ± 0.20
*B. subtilis*	8.0 ± 0.50	25	17.5 ± 0.3	0.78	18.5 ± 0.70	0.78	24.2 ± 0.18	0.097	6.5 ± 0.22
*S. epidermidis*	8.5 ± 0.54	25	13.5 ± 0.5	3.125	15.2 ± 0.33	3.125	16.3 ± 0.40	1.56	6.0 ± 0.20
Gram-negative bacteria									
*P. aeruginosa*	8.2 ± 0.40	25	19.5 ± 0.5	0.39	22.2 ± 0.60	0.195	25.5 ± 0.33	0.097	10.0 ± 0.55
*E. coli*	7.2 ± 0.50	50	13.5 ± 0.77	3.125	14.4 ± 0.50	3.125	17.9 ± 0.17	0.78	7.6 ± 0.65
*K. pneumoniae*	7.0 ± 0.24	50	14.0 ± 0.33	3.125	12.4 ± 0.30	6.25	16.2 ± 0.19	1.56	Nil
*P. vulgaris*	8.0 ± 0.30	25	Nil	100	Nil	100	12.0 ± 0.42	6.25	7.0 ± 0.17
*S. typhi*	7.5 ± 0.15	50	9.2 ± 0.15	12.5	10.2 ± 0.13	6.25	12.9 ± 0.55	6.25	7.3 ± 0.70
*P. mirabilis*	7.0 ± 0.20	50	7.5 ± 0.55	50	13.3 ± 0.55	3.125	12.5 ± 0.50	6.25	12.0 ± 0.20
Unicellular fungi									
*C. albicans*	8.5 ± 0.50	25	15.0 ± 0.25	3.125	15.5 ± 0.20	3.125	17.2 ± 0.20	1.56	7.9 ± 0.16
*C. tropicalis*	8.0 ± 0.25	25	15.1 ± 0.20	3.125	16.6 ± 0.50	1.56	18.0 ± 0.20	0.78	8.0 ± 0.70
LSD	0.33335	–	0.33456	–	0.23235	–	0.45345	–	0.65345

Nil means that no ZOI was detected

*AX* Amoxicillin (antibacterial standard), *NS* Nystatin (antifungal standard), *LSD* Least Significant Differences

In contrast to Zhang et al.’s antibacterial potential results [46], zones of inhibition for *E. coli*, *P. aeruginosa*, and *S. aureus* were determined to be 9.1, 14.5, and 18.0 mm, respectively. For *E. coli* and *P. aeruginosa*, the corresponding MIC and MBC values were 7.8125 and 62.5 μg/mL and 7.8125 and 31.25 μg/mL, respectively. Regarding *S. aureus*, the MIC and MBC were 15.625 and 31.25 μg/mL, respectively. Ag NPs offer superior antibacterial activity against both Gram-positive and Gram-negative bacteria, according to Zhang et al.’s data [46].

Previous research has mostly focused on bacterial cells when examining the impact of light on antimicrobial properties [56, 57]. It has been demonstrated that exposure to light increases the production of reactive oxygen species (ROS), which harms bacterial cells [58]. Light can suppress the growth of *Fusarium oxysporum* (N and F co-doped TiO_2_ NPs) and diminish *C. albicans* biofilms (Ag NPs), according to a current study on light-activated chemically produced NPs [59, 60]. But in addition to affecting how well NPs function, light also promotes the growth of fungi, which in turn causes the expression of many metabolic pathways [61]. In the current investigation, higher quantities of NPs were needed to suppress fungal growth in *C. albicans* treated with Co(OH)_2_ NPs (before calcination) and *C. glabrata* treated with AgNPs before calcination [61]. Further research is necessary since the data point to a possible activation of fungal defense systems in the presence of light.

### Antibiofilm activity of RP-ZnO, RP-CuO, and RP-MgO nanoconjugates

**Fig. 5:**
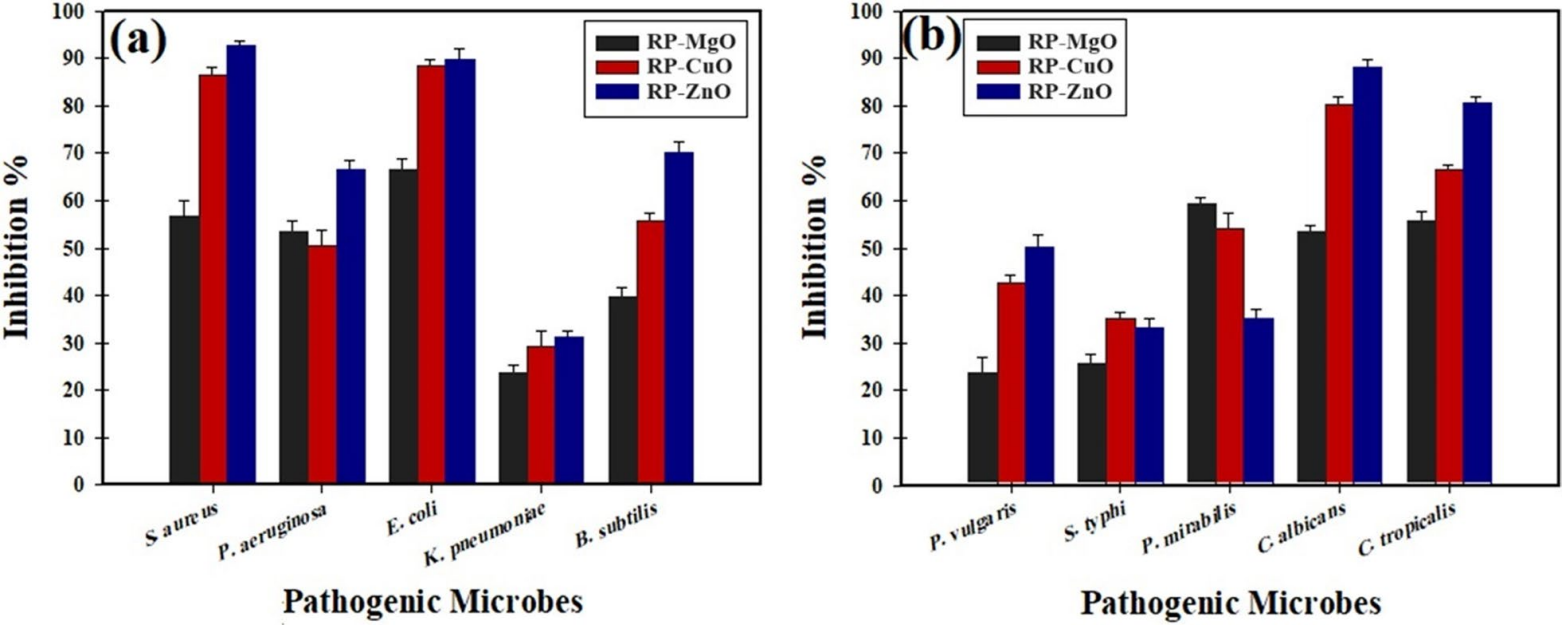
Antibiofilm potential of RP-MgO, RP-CuO, and RP-ZnO nanoconjugates against some bacteria (**a**), and some bacteria, and unicellular fungi (**b**)

Figure 5 presents the recorded results of the antibiofilm potential of the synthesized RP-ZnO, RP-CuO, and RP-MgO nanoconjugates against the chosen bacterial and fungal strains. The highest percent of inhibition of the synthesized RP-MgO, RP-CuO, and RP-ZnO nanoconjugates was recorded against *E. coli* (66.58, 88.58, and 89.58%), and *C. albicans* (53.50, 80.25, and 88.25%), *S. aureus* (56.85, 86.58, and 92.58%), respectively (Fig. 5a and b). The synthesized RP-MgO, RP-CuO, and RP-ZnO nanoconjugates hindered biofilm construction at its irreversible bonding step [62]. However, the exact mechanism of the synthesized samples against biofilm formation remains unconfirmed. The inhibitory percentage variations can be attributed to several factors, including physical properties, antimicrobial action, penetration capabilities, bio-sorption, and interactions between the conjugate and the microbial biofilm [63, 64]. Our results further showed that the synthesized RP-MgO, RP-CuO, and RP-ZnO nanoconjugates were observed to inhibit microbial growth by more than 98%. Similarly, microbial cells failed to produce biofilm due to the inhibition of exopolysaccharide, thereby forming microbial capsules [24].

### Growth curve method

**Fig. 6:**
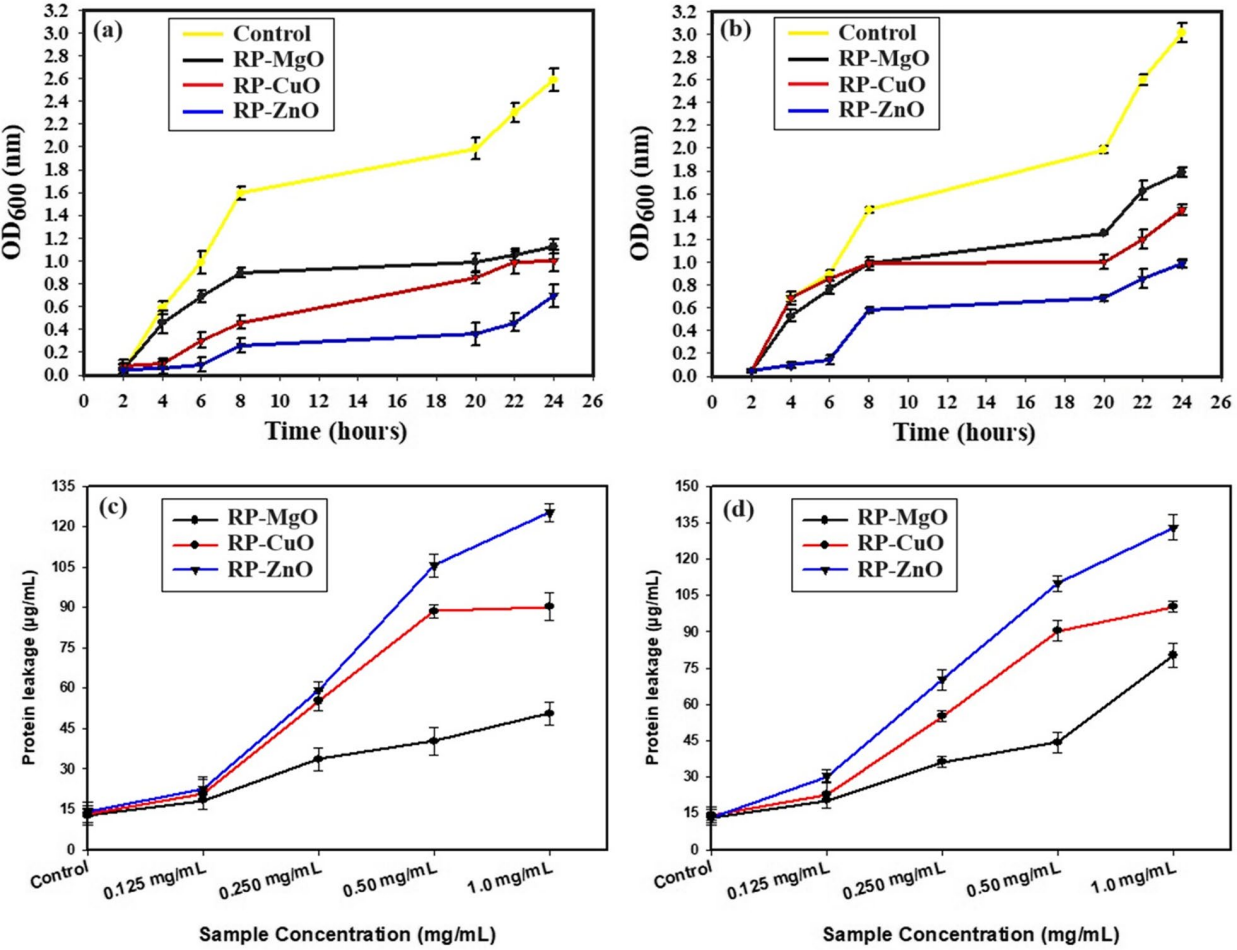
Effect of RP-MgO, RP-CuO, and RP-ZnO nanoconjugates on growth curves of *S. aureus* (**a**) and *E. coli* (**b**), and the protein leakage from cell membranes of *S. aureus* (**c**) and *E. coli* (**d**)

Figure 6a, b presents the effect of the synthesized RP-MgO, RP-CuO, and RP-ZnO nanoconjugates on *S. aureus,* and* E. coli* growth. Our results indicated that *S. aureus *growth in the control sample was fast and the highest value reached 2.58 nm. After adding RP-MgO nanoconjugate, the OD was decreased 1.12. In the case of RP-CuO, the OD value was 1.00. Meanwhile, the value of RP-ZnO was the lowest recording 0.69, indicating growth repression of *S. aureus*. Similarly, *E. coli* growth in the control sample was fast and the highest value reached 3.01. After adding RP-MgO nanoconjugate, the OD was decreased to 1.45. In the case of RP-CuO, the OD value was 1.00. Meanwhile, the value of RP-ZnO was the lowest recording 1.80, indicating growth repression of *E. coli*. Previous reports have characterized the photo-generation of reactive oxygen species on the surface of metal oxide NPs [14, 16, 65]. ZnONPs produce reactive oxygen species, which induce protein oxidation, DNA damage, and lipid peroxidation, effectively killing bacteria without harming other cells. Additionally, the membranes of *S. aureus* and *E. coli* carry a negative charge, while the zinc ions released from ZnONPs have a positive charge. This leads to direct interactions that disrupt DNA replication, protein denaturation, and bacterial cell collapse. The heightened sensitivity of bacteria to ZnONPs may be attributed to the lower stability of the bacterial cell membrane. Another potential reason could be the size, shape, and surface charge of ZnONPs, which enhance their ability to interact with bacteria.

### Bacterial protein leakage evaluation

Results presented in Fig. 6c, d indicated the dose-dependent manner between the applied concentration of the nanoconjugate and the amount of protein; by increasing the nanoconjugate concentration, the protein leakage in both* E. coli* and *S. aureus* was increased. The highest values of protein leakage in *E. coli* after separate treatment with 1.0 mg mL^−1^ of RP-MgO, RP-CuO, and RP-ZnO were 50.58, 90.25, and 125.25 µg mL^−1^, respectively. While, these values were 80.20, 100.25, and 133.02 µg mL^−1^ in *E. coli*. Our results demonstrated the antibacterial potential of the RP-MgO, RP-CuO, and RP-ZnO nanoconjugates suggesting the presence of holes in *E. coli* and *S. aureus* membranes which the bacterial proteins to bleed out from the cell cytoplasm. Our results also suggested that the RP-ZnO nanoconjugate, as the most promising one, had a greater bacterial membrane dissolving ability, thereby altering the permeability, more than the other two nanoconjugates; RP-MgO, and RP-CuO. A plausible explanation for the main cause for the repression of bacterial growth is the effect of the metal and the RP against bacterial membranes [64]. Following these results, previous reports defined the same results after the treatment with NPs [66] where a concentration dependence for the dislodgement in the bacterial membrane suggests leakage of intracellular organelles [67]. In general, leakage developed over time as typical microbial damage, leading to cell collapse due to the loss of cell constituents [68]. It is understood that the synthesized RP-ZnO nanoconjugate initiates its action by adhering to the microbial surface through charge attraction and high surface area. This interaction causes leakage of the bacterial membrane, forming holes and cavities, ultimately halting ion transport within the bacterial cells [64]. Additionally, the generation of reactive oxygen species penetrates the bacterial cell, damaging key cellular organelles and leading to cell death.

### UV light irradiation effect on the RP-ZnO, RP-CuO, and RP-MgO nanoconjugates

**Fig. 7:**
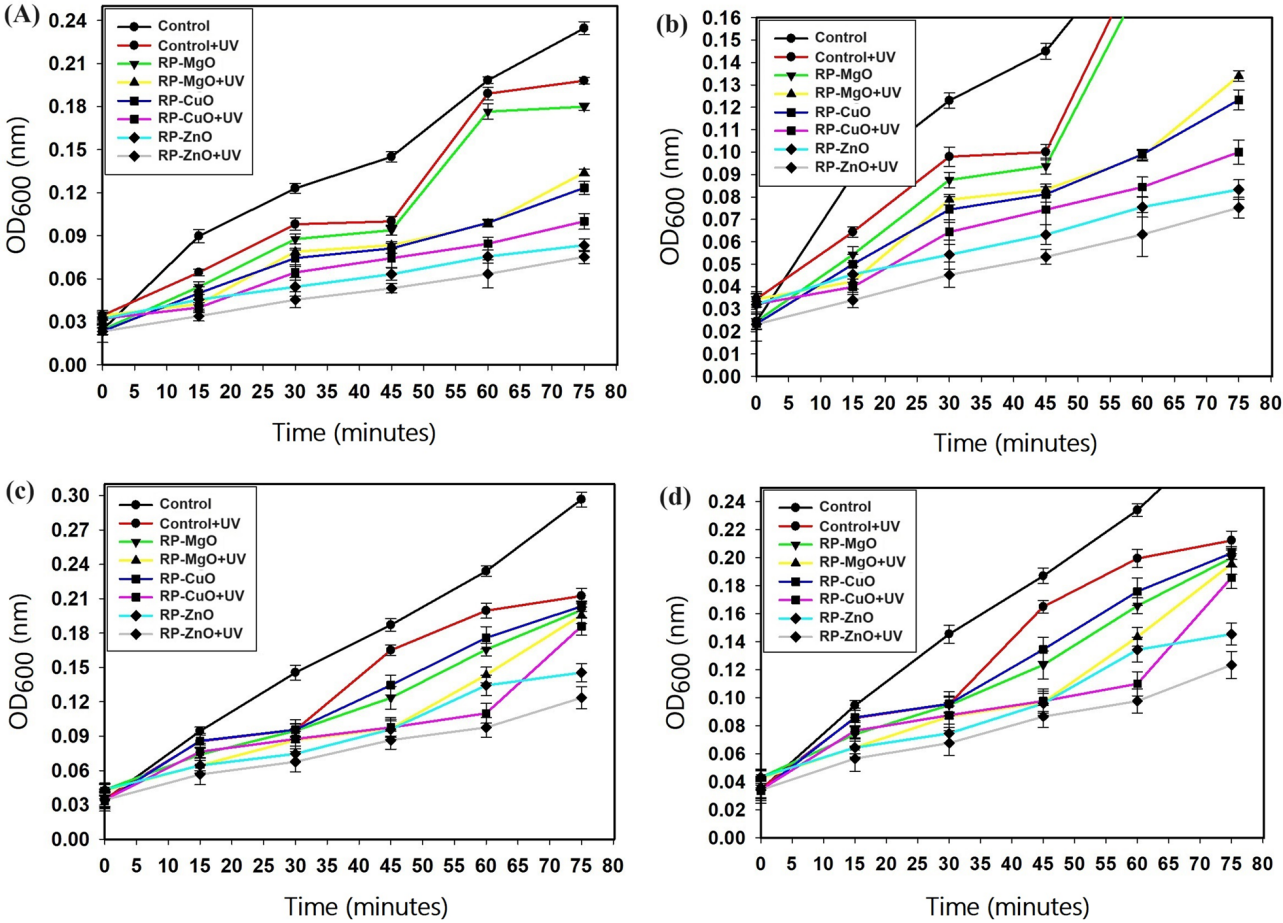
The UV effect on the antibacterial activity of RP-MgO, RP-CuO, and RP-ZnO nanoconjugates against* S. aureus* (**a**), the magnified part (**b**), and* E. coli* (**c**), the magnified part (**d**)

The ability of UV light irradiation to deactivate *S. aureus* and *E. coli* is shown in Fig. 7a–d, respectively, and the degree of sensitivity rises with exposure duration. Throughout the whole display duration, there were favorable effects on the adhesion and proliferation of *E. coli* and* S. aureus* (0 to 75 min with 15 min time increments). After being treated with RP-ZnO nanoconjugate, the growth of *E. coli* and* S. aureus* was significantly suppressed, compared to the untreated comparison study and other nanoconjugates (RP-MgO and RP-CuO). Owing to the deactivation following UV light irradiation, the bacterial growth in the UV experiment ended at the lowest level. It was determined that exposure to UV light irradiation would enhance the possibility of photo-activation of the RP-ZnO nanoconjugate; also, the likelihood of RP-ZnO nanoconjugate was greater than that of RP-MgO, and RP-CuO nanoconjugates. Once activated by UV light irradiation, RP-ZnO nanoconjugate functions as a highly effective disinfectant. ZnONPs were found to absorb photons and have a role in the production of new ROS (O_2_^−^ and H_2_O_2_) as well as active hydroxyl (⋅OH) when O_2_ and H_2_O are present in the atmosphere and/or water states [69]. One way to see microbial decontamination is as reactive oxygen species (H_2_O_2_) interacting with the membranes. After the microbes penetrated, the effective barrier was seen, and the oxidative hydroxyl free radicals remained stable and vigorous [64]. Moreover, UV light irradiation for 80 min of NPs damaged the cell membrane of *E. coli*, indicating that completed disinfection [70, 71].

### Proposed antibacterial reaction mechanism of the synthesized nanoconjugates

**Fig. 8:**
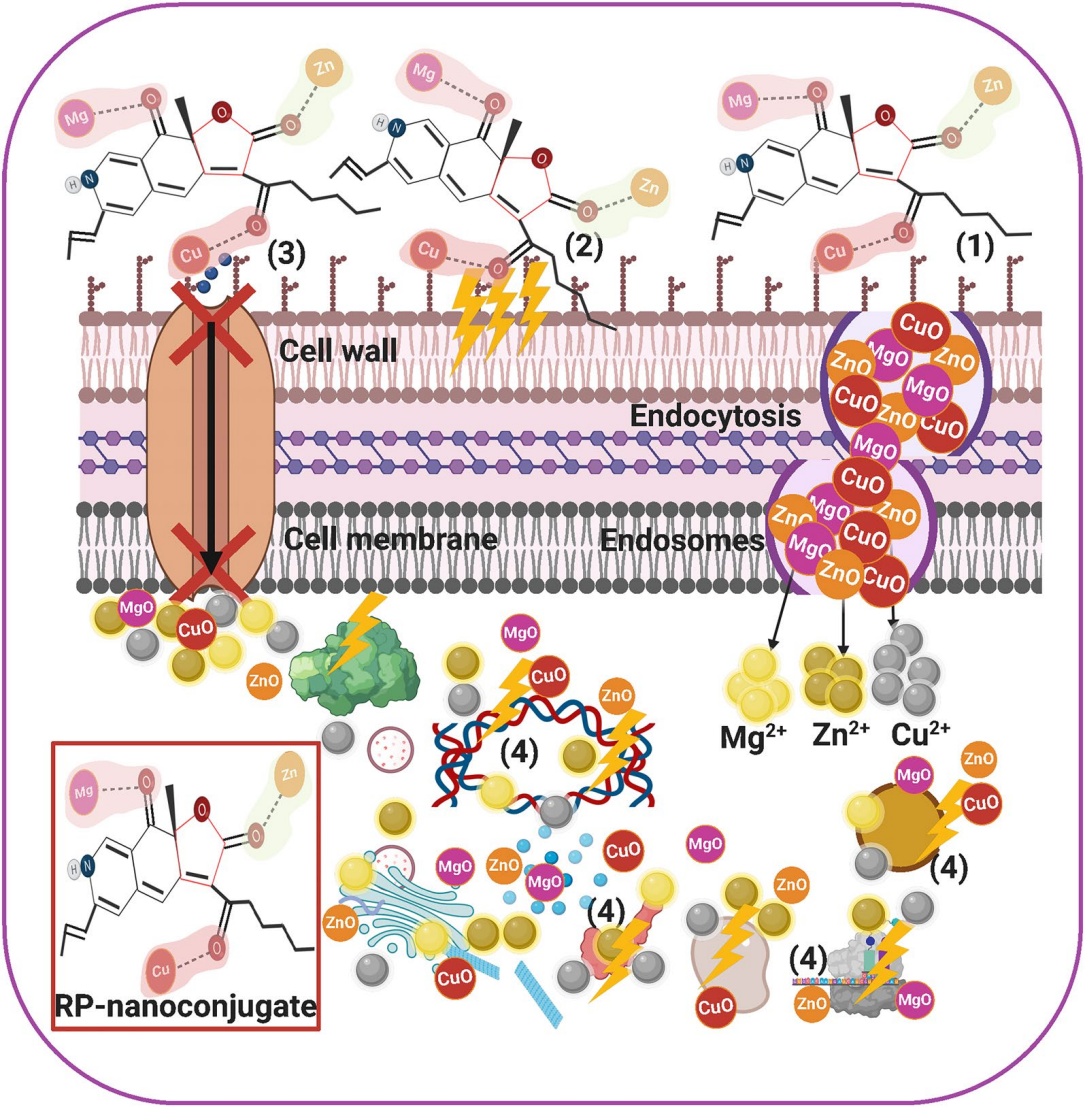
Schematic representation of antimicrobial activity mechanisms (1, 2, 3, and 4) of RP-MgO, RP-CuO, and RP-ZnO nanoconjugates. The figure was created in BioRender.com

Based on the antibacterial activity findings of the prepared nanoconjugates, we conducted more investigation on the mechanism of action of the produced RP-ZnO, RP-MgO, and RP-CuO on the bacterial cell. It is unclear how precisely the prepared nanoconjugates affect cells. The unique methods of action of the prepared nanoconjugates are shown in Fig. 8. These processes include adherence to bacteria’s cell walls and membranes, disruption of intracellular organelles and biomolecules during cell penetration, production of oxidative damage, and modification of signaling cascades [72]. Because of their ability to damage bacterial cells by adhering to essential cellular structural components, especially their SH groups, the produced nanoconjugates are believed to have bactericidal properties [73]. Moreover, they generate reactive oxygen species and free radicals, which damage cell membranes and obstruct respiratory enzymes [74]. Moreover, the produced nanoconjugates produce free radicals, which damage membranes, they are hypothesized to have antibacterial qualities [54]. The produced nanoconjugates can halt bacterial cell division and replication, ultimately resulting in bacterial apoptosis. Strong interactions exist between Zn, Cu, and Mg ions and the thiol groups present in phosphorus-containing bases and enzymes [54]. Collectively, the expected process of the generated nanoconjugates’ interaction with the bacterial cell is shown in Fig. 8. Among these responses are the following ones: RP-metal oxide nanoconjugates have four main effects on bacterial cells: (1) they stick to the outside of the cell and cause membrane failure, endocytosis, the formation of endosomes, and altered transport potential; (2) they produce and increases reactive oxygen species (ROS), which may indicate that the cell wall is starting to weaken; (3) they block ions from passing through the bacterial cell; and (4) they enter the cell and interacts with organelles, like DNA, to alter their functions and cause bacterial cell lysis. RP-metal oxide nanoconjugates may also act as a carrier to efficiently release Zn, Mg, and Cu ions in the cytoplasm and layer, where the presence of a proton motive force may cause the pH to fall below 3.0 and cause the discharge of Zn, Mg, and Cu ions.

## Conclusion

Three novel nanoconjugates were developed using natural RP and gamma irradiation. The synthesized nanoconjugates were characterized by different techniques. The nanoconjugates showed promising antimicrobial potential according to the agar-well diffusion, anti-biofilm, and protein leakage assays. Bacterial growth was significantly suppressed by treatment with UV light-irradiated nanocomposites. Consequently, the three nanoconjugates synthesized in this study may pave the way for their possible applications in different fields, including various biomedical applications.

## Supplementary Information

Supplementary Material 1.

## Data Availability

No datasets were generated or analysed during the current study.
