# Supplementary figures and images for: Novel nanoconjugates of metal oxides and natural red pigment from the endophyte Monascus ruber using solid-state fermentation
Source: Microb Cell Fact. 2024 Sep 29;23:259. doi: 10.1186/s12934-024-02533-8 (PMC11439306; doi:10.1186/s12934-024-02533-8)

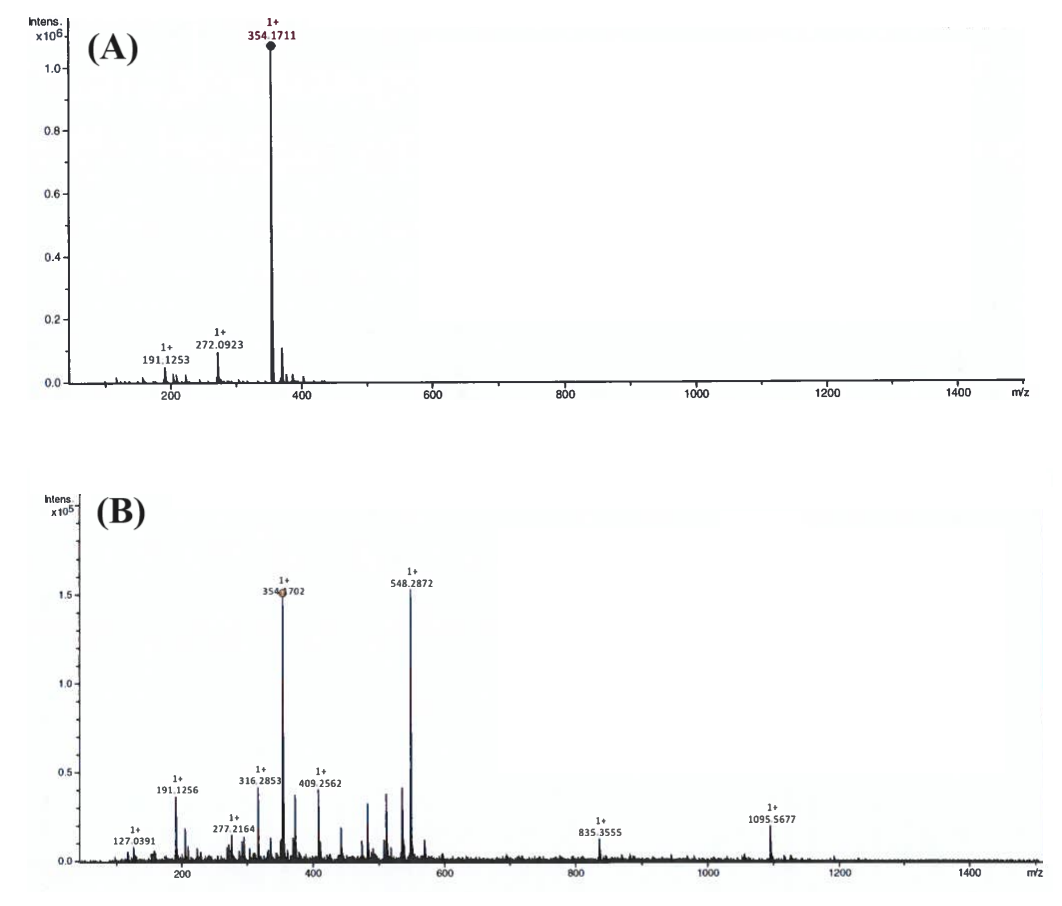

Supplement: Supplementary file 1 — Supplementary Material 1. [file 12934_2024_2533_MOESM1_ESM.tif]
